# A systematic review and net meta-analysis of the effects of different warm-up methods on the acute effects of lower limb explosive strength

**DOI:** 10.1186/s13102-023-00703-6

**Published:** 2023-08-29

**Authors:** F. Y. Li, C. G. Guo, H. S. Li, H. R. Xu, P. Sun

**Affiliations:** College of Physical Education and Sports, Beijing Normal University, North Taipingzhuang Street, Beijing, 100875 China

**Keywords:** Warm-up methods, Explosive lower limb strength, Reticulation meta-analysis, Sprint time, Jump height

## Abstract

**Objective:**

To evaluate the effects of different warm-up methods on the acute effect of lower limb explosive strength with the help of a reticulated meta-analysis system and to track the optimal method.

**Methods:**

R software combined with Stata software, version 13.0, was used to analyse the outcome metrics of the 35 included papers. Mean differences (MD) were pooled using a random effects model.

**Results:**

1) Static combined with dynamic stretching [MD = 1.80, 95% CI: (0.43, 3.20)] and dynamic stretching [MD = 1.60, 95% CI: (0.67, 2.60)] were significantly better than controls in terms of improving countermovement jump height (cm), and the effect of dynamic stretching was influenced by the duration of stretching (I^2^ = 80.4%), study population (I^2^ = 77.2%) and age (I^2^ = 75.6%) as moderating variables, with the most significant effect size for dynamic stretching time of 7–10min. 2) Only dynamic stretching [MD = -0.08, 95% CI: (-0.15, -0.008)] was significantly better than the control group in terms of improving sprint time (s), while static stretching [MD = 0.07, 95% CI: (0.002, 0.13)] showed a significant, negative effect. 3) No results were available to demonstrate a significant difference between other methods, such as foam axis rolling, and the control group.

**Conclusion:**

The results of this review indicate that static stretching reduced explosive performance, while the 2 warm-up methods, namely dynamic stretching and static combined with dynamic stretching, were able to significantly improve explosive performance, with dynamic stretching being the most stable and moderated by multiple variables and dynamic stretching for 7–10min producing the best explosive performance. In the future, high-quality studies should be added based on strict adherence to test specifications.

## Introduction

Warming up as a routine activity before a match or training has been popular in the sports training community for centuries. A scientific warm-up not only helps athletes to become fit but also improves joint mobility and muscle contraction to reduce the incidence of sports injuries [1]. With the development of science and technology, new warm-up methods have emerged, but the opinion remains mixed on which is more suitable for athletes' explosive performance.

Muscle stretch (MS) has received much attention as an important part of the preexercise warm-up [2]. But the effect of stretching on explosive power remains controversial, for example, previous studies have shown that static stretching has a negative effect on subsequent performance [3, 4], while dynamic stretching has a beneficial effect [5], but the opposing views have emerged in recent years [6, 7]. With the advent of combined stretching methods [8], some subjective researcher bias between studies, as well as differences in outcome indicators, leading to some variation in the effect values of intervention results, thus affecting the accuracy of the results. The advent of the foam rolling (FR) technique [9] has led to an increasing number of coaches and athletes promoting this technique and abandoning the original stretching method. Therefore, there remains a lack of clarity regarding which warm-up method is more appropriate for explosive performance, what the dose–effect relationship is, and what the effects of different warm-up methods are.

The recent maturation of reticulated meta-analysis theory and techniques has provided a method for comparison between multiple interventions, and it has become feasible to analyse the effects of different warm-up methods on the acuity of lower limb explosive strength. Based on this fact, this study used Bayesian reticulated meta-analysis to statistically evaluate the effects of different warm-up methods on the acute effect of explosive strength in the population receiving different warm-up methods to suggest the optimal warm-up method and provide some theoretical basis and reference for the development of precompetition or pretraining preparation activity programmes.

## Information and methods

This systematic review was prospectively registered Open Science Framework (INPLASY; DOI:10.37766/inplasy2023.3.0031) and followed guidelines by the Preferred Reporting Items for Systematic Reviews and Meta-Analyses (PRISMA) and Cochrane Handbook.

### Data sources and study selection

Two researchers (FYL and CGG) independently searched Google Scholar, PubMed, Web of Science, Elsevier, Scopus, CNKI and Wanfang databases from January 2000 to December 2022, with the language limited to Chinese and English. Guidelines from Preferred Reporting Items for Systematic Reviews and Meta-Analyses (PRISMA) [10] were followed throughout. Standard Boolean operators (AND, OR) were used to concatenate the search terms. The search string used in seven electronic databases is displayed in Table 1. This was supplemented by a manual search to trace references for inclusion in order to ensure the comprehensiveness of the included literature.

**Table 1:** Subject search

Group	Search strategy
Group 1	[Title/Abstract] “Static-Stretching” OR “Dynamic- Stretching” OR “Ballistic-Stretching” OR “Proprioceptive Neuromuscular Facilitation” OR “PNF-Stretching” OR “Static-Dynamic Stretching” OR “Combine of Stretching” OR “Foam Rolling” OR “Different stretching methods”
Group 2	[Title/Abstract] “Lower Extremity Explosive Performance” OR “Vertical Jump” OR “Countermovement Jump” OR “Sprint” OR “Speed”
Group 3	[Title/Abstract] “Acute Efects”

### Inclusion and exclusion criteria

The criteria for inclusion in the literature were based on the PICOS principles of evidence-based medicine, considering 5 aspects: study subjects, interventions, control group, study outcomes and study design as follows: 1) study subjects – participants aged ≥ 14 years, free of other injuries and disease conditions prior to the intervention; 2) interventions – the intervention methods used in the experimental group included static stretching, dynamic stretching, ballistic stretching, proprioceptive neuromuscular facilitation, static-dynamic stretching, foam rolling; 3) control group – no warm-up exercise or light aerobic running; 4) study outcomes – the jump index was selected as countermovement jump (CMJ), the sprint index was selected as the 20-m sprint and the 30-m sprint; and 5) study design – due to the specificity of the warm-up method intervention (short intervention time), the study design included RCTs and an own before-and-after controlled trial design. There were no significant differences between the experimental and control groups at baseline.

The exclusion criteria were as follows: 1) literature that did not meet the inclusion criteria; 2) review literature or dissertations; 3) literature that was not in English or Chinese; and 4) literature with incomplete data on outcome indicators, resulting in data that could not be extracted.

### Literature screen and data extraction

First, relevant literature was searched in various databases according to the developed literature search strategy. Then, the literature was uniformly imported into Endnote X9 software for deweighting, followed by an initial screening of literature titles, abstracts and keywords by two researchers (FYL and CGG) using an independent double-blind approach according to the above inclusion criteria. Finally, qualitative and quantitative analyses were conducted on the screened eligible literature. The literature that met the criteria was independently extracted and included the following 3 aspects: (1) general information – first author, year of publication; (2) intervention characteristics – sample size, study population, intervention content, intervention duration; and (3) outcome indicators – countermovement jump (CMJ), 20-m sprint and 30-m sprint indicators were selected.

### Risk of bias evaluation

Based on the characteristics of the included literature, this study used the Methodological Index for Non-randomized Studies (MINORS) risk of bias tool [11] to assess the quality of the literature in 12 domains (clear statement of study purpose, consistency of patients included, collection of expected data, whether the outcome indicators reflected the study purpose, whether the outcome indicators were objective, adequacy of follow-up time, failure rate less than 5%, whether sample size was estimated, appropriateness of control group selection, synchronisation of control groups, comparability of baseline between groups, appropriateness of statistical analysis) to evaluate the quality of the literature, with a score of 0–2 for each domain and a maximum score of 24. Two researchers (HSL and HRX) then independently examined each full-text manuscript against the eligibility criteria. Any disagreements were resolved through discussion and consultation with a third author (PS).

### Statistical analyses

Bayesian MeSH meta-analysis was performed using R software running the gemtc package in the R studio environment in conjunction with Stata software, version 13.0. The outcome indicators in this study were continuous variables, and the meandifference (MD) and 95% confidence interval (95% CI) were used as effect size indicators. Each model was set using four Markov chains for initial values, and the number of iterations was set at 20,000, with the first 5000 used for annealing. Model inconsistency was diagnosed using R software, and Brooks-Gelman-Rubin diagnostic plots were plotted to quantitatively evaluate the convergence of the models. Local inconsistency was tested using the nodal separation method. Finally, thresholds for the interpretation of I^2^ were in line with Cochrane recommendations: 0%–40% (“might not be important”), 30%–60% (“may represent moderate heterogeneity”), 50%–90% (“may represent substantial heterogeneity”), and 75%–100% (“considerable heterogeneity”). The network relationships were mapped using Stata software, version 13.0, and analysed for risk of publication bias; the metrics were ranked by surface under the cumulative ranking (SUCRA), where 0 ≤ SUCRA ≤ 100%, 100% representing the most effective warm-up method and 0 the worst and least effective. Finally, subgroup analyses were conducted to explore the effects of moderating variables.

## Results

### Results of the literature screening

**Fig. 1:**
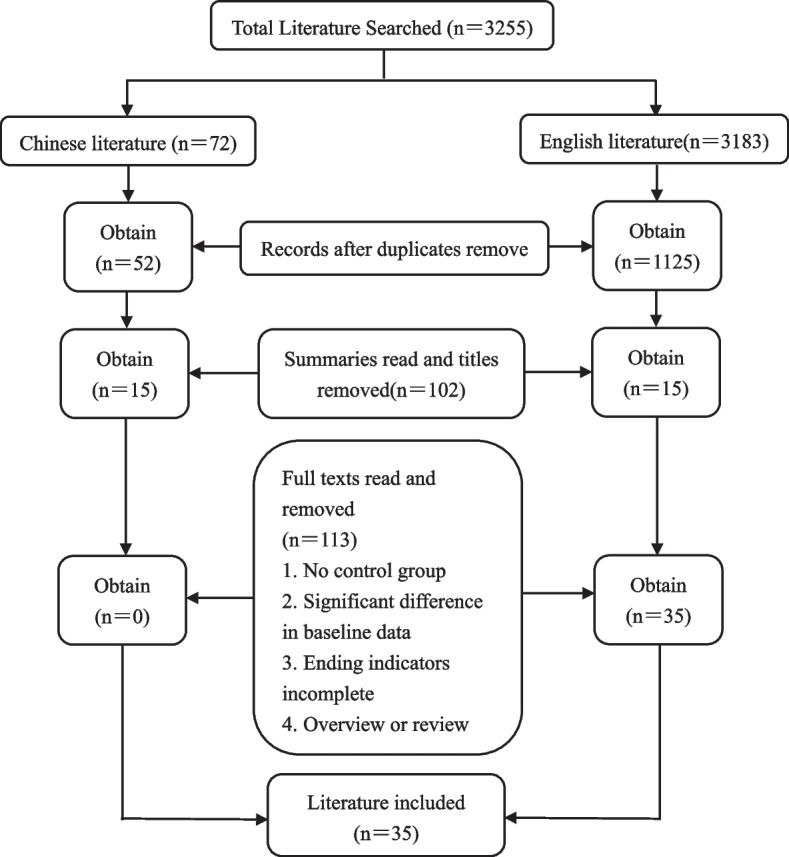
Flow diagram regarding article selection for meta-analysis

A total of 3255 relevant documents were retrieved from the seven selected search libraries, including 72 documents in Chinese and 3183 documents in English. A total of 2078 duplicate publications were excluded, 1029 documents were excluded on the basis of reading the titles and abstracts, and 110 documents were excluded on the basis of reading the full texts. Thirty-five documents were finally included in the literature in English, and the screening flow chart is shown in Fig. 1.

### Basic characteristics of the included studies

Among the included papers, all articles were published in English [12–46]. All original studies included in the trial were RCTs and had a before-and-after controlled trial design. The interventions provided to the experimental group included static stretching, dynamic stretching, ballistic stretching, PNF stretching, static-dynamic stretching and foam rolling, while the interventions provided to the control group were mild aerobic exercise or no sports. The basic characteristics of the included studies are shown in Table 2.

**Table 2:** Basic characteristics of the included studies

First author	Year	Sample size		Age (years)	Intervention methods		Time (min)	Indicators
		Experimental group	Control group		Experimental group	Control group		
Ari(1) [12]	2021	11 11	11	24.4 ± 5.1	Static Stretching	No sports	8 min	a
					Dynamic Stretching			
Fortier [13]	2013	15	15	22.8 ± 2.0	Static Stretching	No sports	4.5 min	a
Paradisis [14]	2014	47 47	47	14.6 ± 1.7	Static Stretching Dynamic Stretching	No sports	6 min	a, b
Loughran [15]	2017	8	8	18–30	Static Stretching	No sports	10 min	a, b
		8			Static-Dynamic Stretching		20 min	
Ryan [16]	2014	26	26	22.2 ± 1.3	Dynamic Stretching	No sports	6.7 min	a
Fletcher(1) [17]	2010	24	24	21.0 ± 0.3	Dynamic Stretching	No sports	NR	a
Curry [18]	2009	24	24	26.0 ± 3.0	Static Stretching	Mild aerobic	10 min	a
		24			Dynamic Stretching			
Nagle [19]	2010	14	15	18–24	Static Stretching	No sports	12 min	a
		13			Dynamic Stretching			
Baumgart [20]	2019	20	20	26.6 ± 2.7	Foam Rolling	No sports	10 min	a
Henning [21]	2019	19	19	M:21.5 ± 1.8	Foam Rolling	No sports	15 min	a
				W:20.2 ± 1.5				
Franco [22]	2019	15	15	EG:24.1 ± 4.2	Foam Rolling	Mild aerobic	8 min	a
				CG:25.0 ± 4.7				
Kopec [23]	2017	20	20	22.5 ± 4.0	Dynamic Stretching	No sports	3 min	a
		20			Foam Rolling			
Oliveira [24]	2017	12	12	17.7 ± 0.9	Ballistic Stretching	No sports	15 min	a, b
		12			PNF Stretching			
Unick [25]	2005	16	16	19.2 ± 1.0	Static Stretching	No sports	6 min	a
		16			Ballistic Stretching			
Kruse [26]	2013	11	11	20.0 ± 1.6	Static Stretching	No sports	7 min	a
		11			Dynamic Stretching			
Morrin [27]	2013	10	10	27.0 ± 5.0	Static Stretching	No sports	8 min	a
		10			Dynamic Stretching			
		10			Static Dynamic Stretching			
Yildiz [28]	2020	35	35	23.6 ± 1.3	Static Stretching	Mild aerobic	NR	a
Chaouachi [29]	2010	22	22	20.6 ± 1.2	Static Stretching	No sports	10 min	a, c
		22			Dynamic Stretching			
		22			Static Dynamic Stretching			
Christensen [30]	2008	68	68	20.5 ± 1.4	Dynamic Stretching	Mild aerobic	NR	a
		68			PNF Stretching			
Fletcher(2) [31]	2010	27	27	20.5 ± 2.2	Static Stretching	Mild aerobic	6 min	a, b
		27			Dynamic Stretching			
Pagaduan [32]	2012	29	29	19.4 ± 1.1	Static Stretching	No sports	7 min	a
		29			Dynamic Stretching			
		29			Static-Dynamic Stretching			
Byrne [33]	2014	29	29	20.8 ± 4.4	Dynamic Stretching	Mild aerobic	11 min	b
Bafghi [34]	2013	15	15	24.7 ± 4.6	Static Stretching	No sports	NR	a
		15			Dynamic Stretching			
Shi Huang [35]	2022	14	14	22.6 ± 1.7	Dynamic Stretching	No sports	8 min	b
Baskurt [36]	2017	15	15	22.9 ± 1.3	Static Stretching	No sports	NR	b
Ari(2) [37]	2021	8	8	15.4 ± 1.1	Static Stretching	No sports	8 min	a
		8			Dynamic Stretching			
		8			Static-Dynamic Stretching			
Dallias [38]	2019	26	26	22.4 ± 3.7	Dynamic Stretching	No sports	NR	b
Utku [39]	2017	12	12	15.0 ± 0.5	Static Stretching	No sports	14 min	b
		12			PNF Stretching			
		12			Ballistic Stretching			
Jaggers [40]	2008	20	20	24.8 ± 3.0	Dynamic Stretching	No sports	NR	a
		20			Ballistic Stretching			
Vetter [41]	2007	26	26	M:21.7 ± 1.2	Static Stretching	Mild aerobic	NR	c
		26		W:22.3 ± 1.6	Dynamic Stretching			
Gelen [42]	2010	26	26	23.3 ± 3.2	Static Stretching	No sports	10 min	c
		26			Dynamic Stretching		10 min	
		26			Static-Dynamic Stretching		20 min	
Nelson [43]	2005	16	16	M:21.0 ± 2.0 W:19.0 ± 1.0	Static Stretching	No sports	NR	b
Adam [44]	2008	10	10	18–29	Static Stretching	Mild aerobic	NR	c
Marinho [45]	2017	16	16	22.0 ± 1.6	Static Stretching	No sports	8–10 min	b
		16			Dynamic Stretching			
Perrier [46]	2011	21 21	21	24.4 ± 4.5	Static Stretching Dynamic Stretching	No sports	13.8 min	a

“NR” indicates not reported

“a” indicates countermovement jump (CMJ) heigh(cm)

“b” indicates 20-m sprint time; “c” indicates 30-m sprint time(s)

### Results of the methodological quality evaluation

The 2 researchers (HSL and HRX) scored each of the 12 domain entries according to the (Methodological Index for Non-randomized Studies, MINORS) scale, and the threshold of disagreement was referred to another researcher (PS) for judgement, resulting in an overall final score for the literature, as shown in Table 3, from which the results of the literature quality evaluation found that the quality scores of the literature for all included studies were relatively high, with risky entries occurring in the use of blinding and whether sample sizes were estimated.

**Table 3:** Results of the methodological quality evaluation

First author	1	2	3	4	5	6	7	8	9	10	11	12	Scores
Ari(1) [12]	2	2	2	2	0	2	2	0	2	0	2	2	18
Fortier [13]	2	2	2	2	0	2	2	0	0	0	2	2	16
Paradisis [14]	2	2	2	2	0	2	2	0	1	0	2	2	17
Loughran [15]	2	2	2	2	1	2	2	0	2	2	2	2	21
Ryan [16]	2	2	2	2	1	2	2	0	0	0	2	2	17
Fletcher(1) [17]	2	2	2	2	0	2	2	0	0	0	1	2	15
Curry [18]	2	2	2	2	1	2	2	0	2	0	2	2	19
Nagle [19]	2	2	2	2	0	2	2	0	1	2	2	2	19
Baumgart [20]	2	2	2	2	0	2	2	0	2	0	2	2	18
Henning [21]	2	2	2	2	0	2	2	0	0	0	2	2	16
Franco [22]	2	2	2	2	0	2	2	0	2	2	2	2	20
Kopec [23]	2	2	2	2	0	2	2	0	0	0	2	2	16
Oliveira [24]	2	2	2	2	0	2	2	0	1	0	2	2	17
Unick [25]	2	2	2	2	0	2	2	0	1	0	2	2	17
Kruse [26]	2	2	2	2	0	2	2	0	2	0	2	2	18
Morrin [27]	2	2	2	2	0	2	2	0	2	0	2	2	18
Yildiz [28]	2	2	2	2	0	2	2	0	1	0	1	2	16
Chaouachi [29]	2	2	2	2	0	2	2	0	1	0	2	2	17
Christensen [30]	2	2	2	2	0	2	2	0	0	0	1	2	15
Fletcher(2) [31]	2	2	2	2	0	2	2	0	2	0	2	2	18
Pagaduan [32]	2	2	2	2	0	2	2	0	0	0	2	2	16
Byrne [33]	2	2	2	2	0	2	2	0	2	0	2	2	18
Bafghi [34]	2	2	2	2	0	2	2	0	2	0	1	2	17
Shi Huang [35]	2	2	2	2	0	2	2	0	2	0	2	2	18
Baskurt [36]	2	2	2	2	0	2	2	0	1	2	1	2	18
Ari(2) [37]	2	2	2	2	0	2	2	0	2	0	2	2	18
Dallias [38]	2	2	2	2	0	2	2	0	2	0	1	2	17
Utku [39]	2	2	2	2	0	2	2	0	0	0	2	2	16
Jaggers [40]	2	2	2	2	0	2	2	0	1	0	1	2	16
Vetter [41]	2	2	2	2	0	2	2	0	1	0	1	2	16
Gelen [42]	2	2	2	2	0	2	2	0	2	0	2	2	18
Nelson [43]	2	2	2	2	0	2	2	0	1	0	1	2	16
Adam [44]	2	2	2	2	0	2	2	0	2	0	1	2	17
Marinho [45]	2	2	2	2	0	2	2	0	1	0	2	2	17
Perrier [46]	2	2	2	2	0	2	2	0	2	0	2	2	18

“1” indicates that the purpose of the study was clearly given; “2” indicates the consistency of the patients included; “3” indicates the expected data collection; “4” indicates whether the outcome indicators reflected the purpose of the study; “5” indicates whether the trial was blinded; “6” indicates whether the follow-up period was adequate; “7” indicates whether the loss of follow-up rate was less than 5%; “8” indicates whether the sample size was estimated; “9” indicates whether the selection of the control group was appropriate; “10” indicates whether the control groups indicated whether the control groups are synchronised; “11” indicates whether the baselines were comparable between groups; “12” indicates whether the statistical analysis was appropriate

### Results of the reticulated meta-analysis

#### Network relations map

**Fig. 2:**
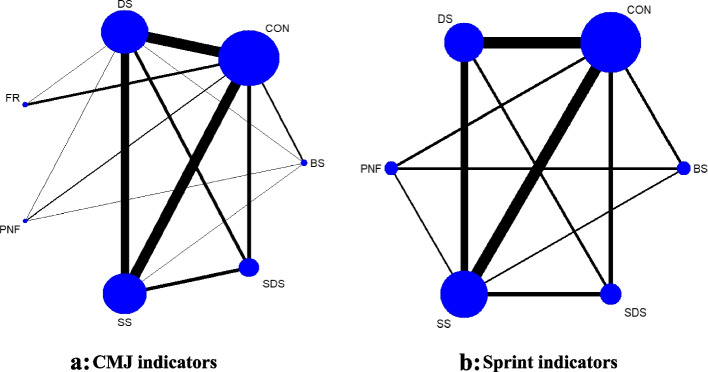
Network diagram of the relationship between different warm-up methods on the explosive strength indicators of the lower limbs. Note: “SS” indicates static stretching, “DS” indicates dynamic stretching, “BS” indicates ballistic stretching, “PNF” indicates PNF stretching, “FR” indicates foam rolling, “SDS” indicates static-dynamic stretching, “CON” indicates control group, The unit of measurement of CMJ indicators is centimeter(cm), The unit of measurement of Sprint indicators is seconds(s)

A total of 25 studies in the overall included literature used the CMJ test, and 15 studies used the sprint test to evaluate the effect of different warm-up methods on the acute impact of lower limb explosive strength. The reticulated relationship graphs generally show a star-like structure centred on the control group, and all formed a triangular closed loop, as shown in Fig. 2. These figures show that there is evidence of both direct and indirect comparisons between different intervention methods in the lower limb explosive strength test; therefore, the basic conditions for a reticulated meta-analysis are present. From the figure, it can be seen that the thickest line between DS, SS and the control group represents the most direct comparison studies, reflecting from the side that the 2 are the most controversial warm-up methods in current sports training, and their mechanism of action on explosive strength has yet to be clarified.

### Overall consistency test and model convergence diagnosis

#### Overall consistency test

The reticulated meta-analysis is based on the assumption of consistency. First, we must judge whether the data are consistent or not, record the DIC_1_ value under the consistency model fit in R software, and then record the DIC_2_ value under the inconsistency model fit to judge the global consistency. If the difference between the two is less than five, the data are generally consistent.

CMJ indicators: DIC_1_ = 105.316 under the consistency model fit was recorded in R software, and then DIC_2_ = 108.412 under the inconsistency model fit was recorded, with a difference less than five, indicating that there was no inconsistency between the intervention methods under direct and indirect comparisons and that the consistency model could be used for analysis.

Sprint indicator: DIC_1_ = 79.577 under the consistency model fit was recorded in R software, and then DIC_2_ = 79.969 under the inconsistency model fit was recorded, with a difference less than five, indicating that the intervention modality was not inconsistent under direct and indirect comparisons and that the consistency model could be used for analysis.

### Model convergence diagnosis

**Fig. 3:**
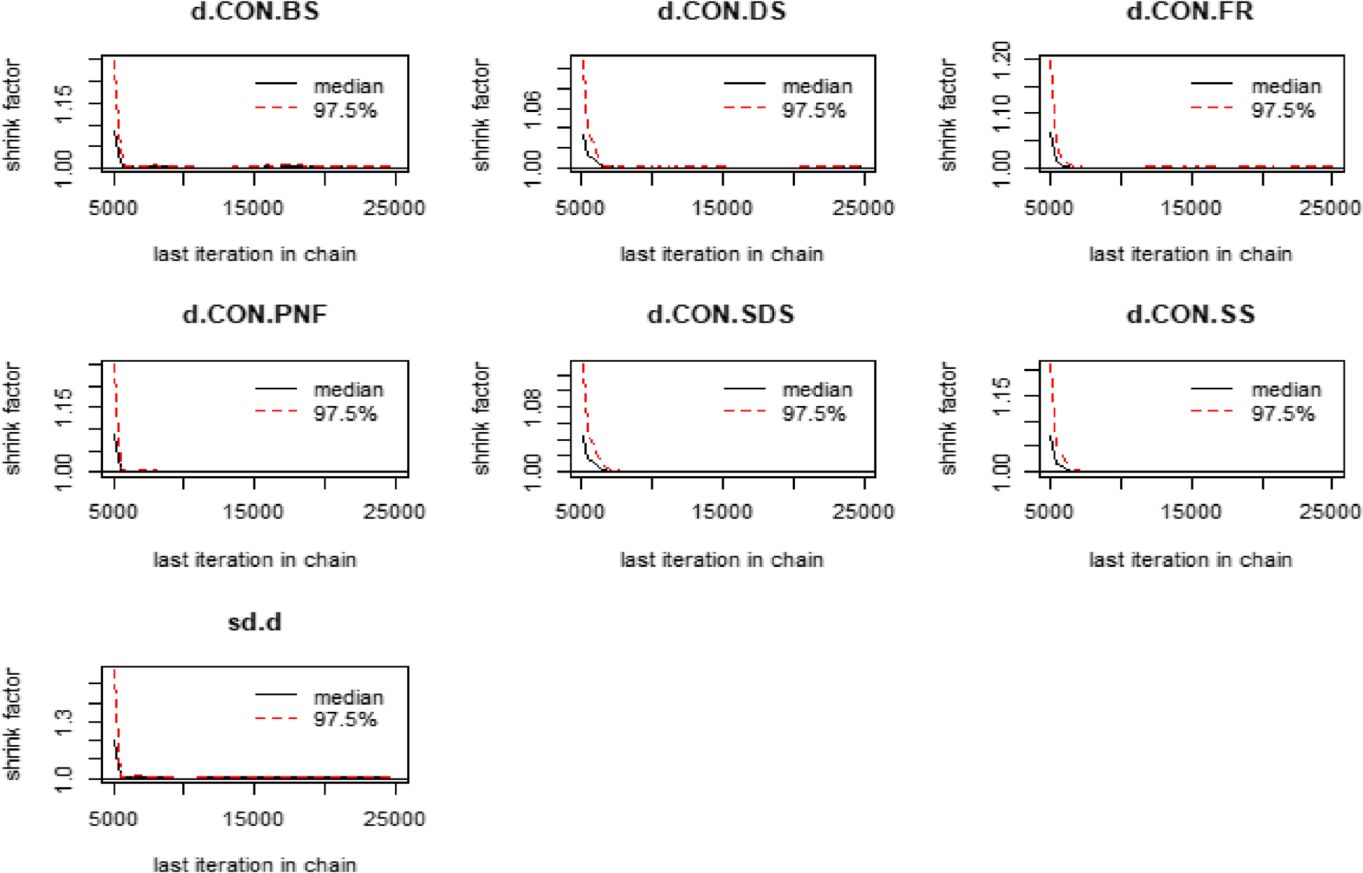
Brooks-Gelman-Rubin diagnostic chart

The Brooks-Gelman-Rubin diagnostic map was used to quantify the degree of convergence of the diagnostic model, as shown in Fig. 3. The results of the images show that the median value of the reduction factor, 97.5% of the reduction factor and the potential scale reduction factor (PSFR) of this study's model are close to one after iterative calculation, and the model converges strongly, proving that the results of the mesh meta-analysis are reliable.

### Direct meta-analysis comparison of results

The study was conducted by plotting direct comparison forest plots to analyse the effects of different warm-up methods on the explosive power of the lower limbs and, by examining at whether the 95% CI crossed the 0 scale, to determine whether the difference was statistically significant.

#### CMJ indicators

**Fig. 4:**
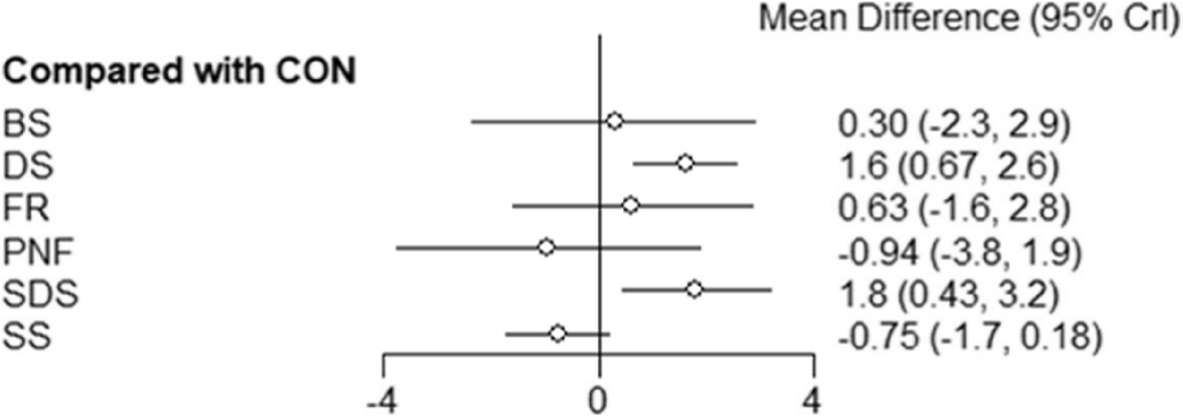
CMJ(cm) indicator forest chart

The results of the reticulated meta-analysis of CMJ(cm) indicators for direct comparison between each warm-up method and the control group are shown in Fig. 4. The acute effects of SDS [MD = 1.80, 95% CI: (0.43, 3.20)], DS [MD = 1.60, 95% CI: (0.67, 2.60)] on CMJ indicators were superior to those of the control group. There was no evidence that BS [MD = 0.30, 95% CI: (-2.30, 2.90)], FR [MD = 0.63, 95% CI: (-1.60, 2.80)], PNF [MD = -0.94, 95% CI: (-3.80, 1.90)], or SS [MD = -0.75, 95% CI: (-1.70, 0.18)] were significantly different from the control group.

#### Sprint indicators

**Fig. 5:**
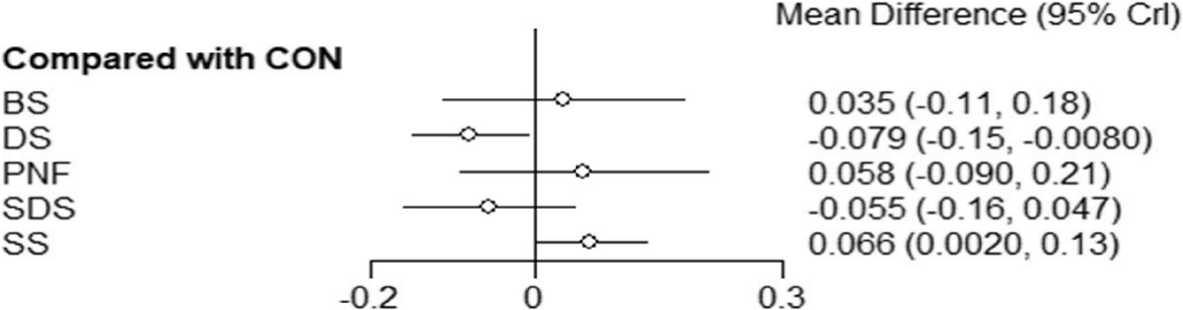
Sprint(s) indicator forest chart

The results of the net meta-analysis of sprint(s) metrics for direct comparison between each warm-up method and the control group are shown in Fig. 5. The acute effect of DS [MD = -0.08, 95% CI: (-0.15, -0.008)] on sprint metrics was better than that of the control group, while the acute effect of SS [MD = 0.07, 95% CI: (0.002, 0.13)] on sprint metrics was less than that of the control group. There was no evidence of significant differences among BS [MD = 0.04, 95% CI: (-0.11, 0.18)], PNF stretch [MD = -0.06, 95% CI: (-0.09, 0.21)], SDS [MD = -0.06, 95% CI: (-0.16, 0.05)] and controls.

### Local consistency check

The local inconsistency test (local inconsistency) was performed by the node-splitting method to test whether there was a difference between the results of direct and indirect comparisons when there was a closed loop between the direct and indirect comparisons, and if the difference was not statistically significant, the results of direct and indirect comparisons were considered to be consistent.

#### CMJ indicators

As shown in Table 4, in all closed loops, the differences between direct and indirect comparisons were not significantly different between the groups (*P* > 0.05), and overall, there was good agreement between direct and indirect interventions where closed loops existed.

**Table 4:** CMJ(cm) indicator node splitting method test

Intervention methods	Direct MD(95%CI)	Indirect MD(95%CI)	Network MD(95%CI)	P
DS vs. BS	0.24(-7.50, 8.00)	1.70(-1.30, 4.60)	1.40(-1.30, 4.10)	0.73
PNF vs. BS	-2.10(-6.20, 2.00)	0.48(-4.90, 5.80)	-1.20(-4.50, 2.10)	0.46
SS vs. BS	0.20(-4.50, 4.90)	-1.50(-5.00, 1.90)	-1.00(-3.70, 1.70)	0.56
FR vs. DS	-0.66(-8.30, 7.00)	-1.00(-3.60, 1.50)	-1.00(-3.40, 1.40)	0.92
PNF vs. DS	-0.08(-4.90, 4.70)	-4.20(-8.10, 0.25)	-2.60(-5.50, 0.29)	0.19
SS vs. DS	-2.30(-3.30, -1.40)	-0.85(-2.60, 4.30)	0.17(-1.20, 1.60)	0.56
SDS vs. DS	-0.28(-1.90, 1.40)	0.85(-2.60, 4.30)	0.17(-1.20, 1.60)	0.56

#### Sprint indicators

As shown in Table 5, the differences between direct and indirect comparisons were not significantly different between the groups in all of the closures (*P *> 0.05), and overall, there was good agreement between direct and indirect interventions for the closures.

**Table 5:** Sprint(s) indicator node splitting method test

Intervention methods	Direct MD(95%CI)	Indirect MD(95%CI)	Network MD(95%CI)	P
SS vs. BS	0.03(-0.23, 0.29)	0.03(-0.19, 0.25)	0.03(-0.12, 0.19)	0.99
SS vs. DS	0.18(0.06, 0.29)	0.09(-0.09, 0.27)	0.14(0.07, 0.23)	0.38
SDS vs. DS	0.07(-0.03, 0.16)	0.04(-0.09, 0.16)	0.02(-0.09, 0.13)	0.68
SS vs. PNF	0.001(-0.26, 0.26)	0.01(-0.21, 0.25)	0.01(-0.15, 0.17)	0.95

### Cumulative probability ranking results

The cumulative probability ranking diagram and SUCRA values were used to comprehensively evaluate the order of merit of different warm-up methods on the acute effects of lower limb explosive strength to screen for the best warm-up method.

#### CMJ indicators

**Fig. 6:**
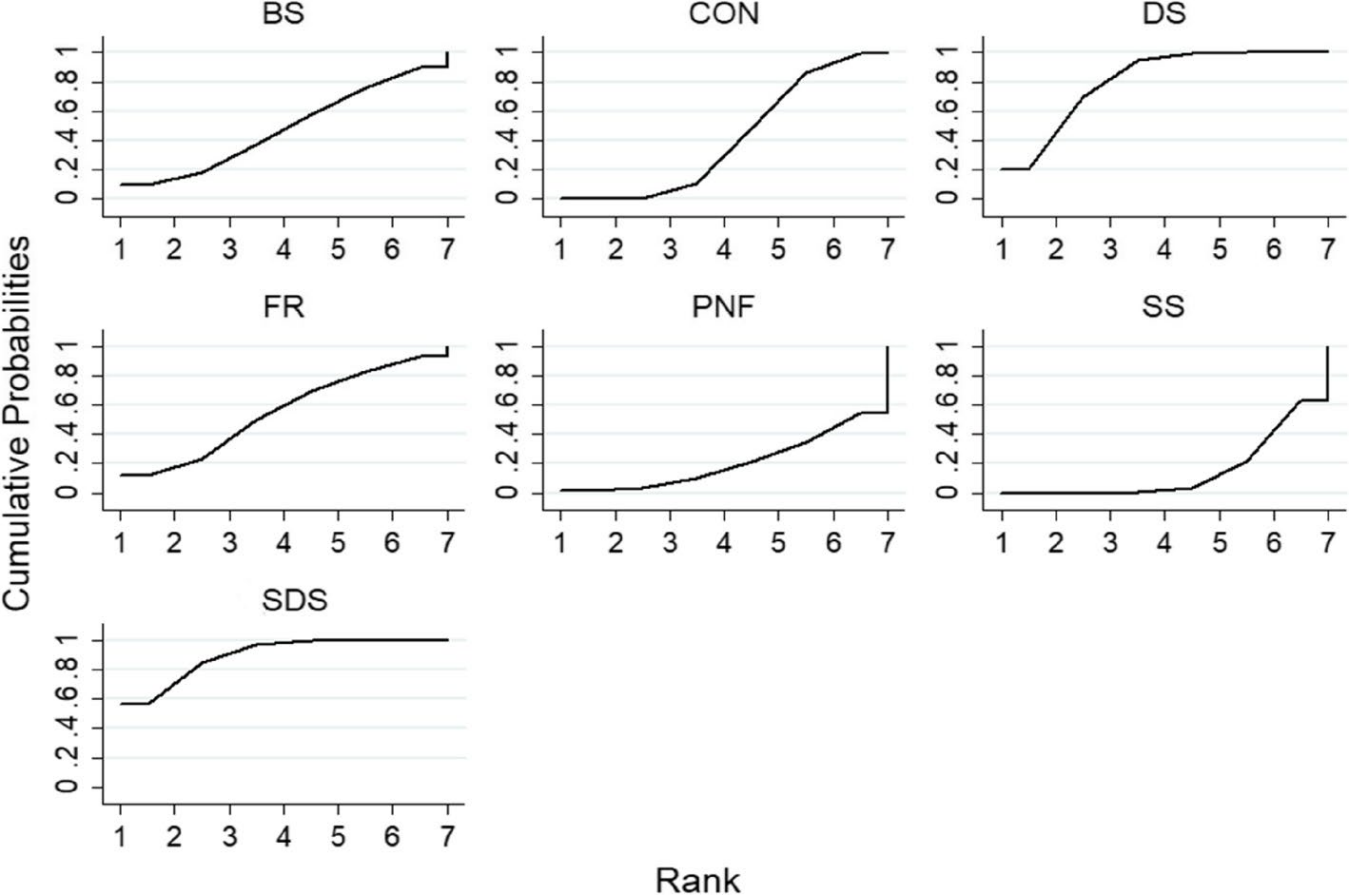
Cumulative ranking probability graph for CMJ indicators

The results of the ranking of the effects of different warm-up methods on the CMJ index are shown in Fig. 6. The results show that SDS (87.6%) > DS (83.5%) > FR (55.5%) > BS (48.1%) > CON (40.2%) > PNF stretch (19.5%) > SS (15.6%).

#### Sprint indicators

**Fig. 7:**
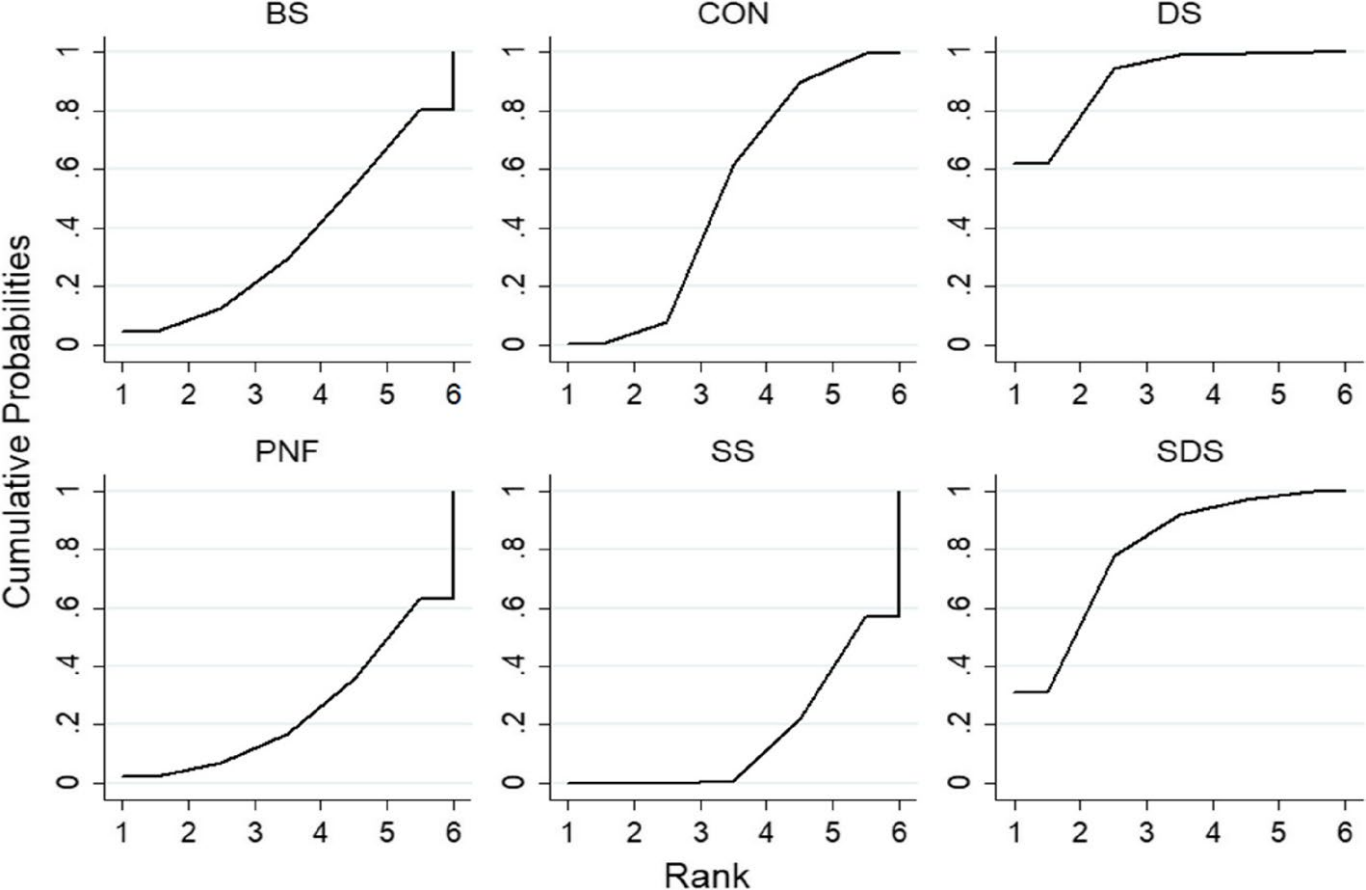
Cumulative ranking probability chart for sprint indicators

The results of the ranking of the effects of different warm-up methods on sprint indices are shown in Fig. 7. The results show that DS (91.1%) > SDS (79.6%) > CON (51.9%) > BS (36.4%) > PNF stretch (25.0%) > SS (16.1%).

### Publication bias analysis

**Fig. 8:**
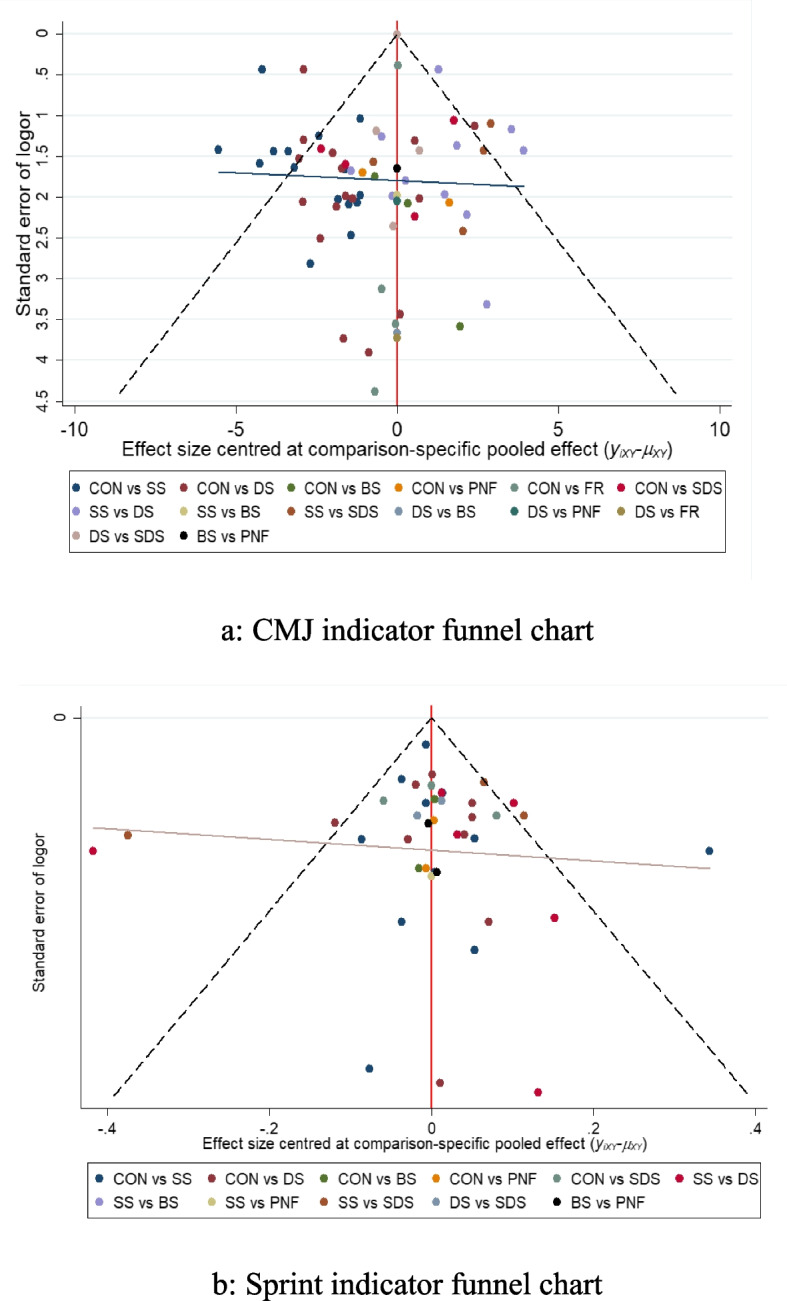
Funnel plot of lower limb explosive strength-related indicators

The 35 included studies were tested for risk of publication bias, and the funnel plot is shown in Fig. 8. The results of the image show that the majority of studies had effect sizes concentrated at the top of the funnel plot, but given that there are still a few studies located at the outside and bottom of the funnel plot, it suggests that there might be a slight publication bias, as well as a small sample effect in this network of studies.

### A test of the moderating effect of dynamic stretching on explosive power

Considering the outcomes together, DS produced the most consistent effect on the acute effect of explosive strength compared to other warm-up methods, and significant heterogeneity in the results of comparisons between DS and controls was found through R software analysis. Further analysis using Stata software yielded the results of the overall effect test, as shown in Table 6, reflecting the possibility of the existence of potential moderating variables. In light of this finding, a moderating effect test was conducted on the 17 DS studies in the total included literature to examine the roles of six moderating variables in the effects of DS on the acute effect of lower limb explosive strength, as shown in Table 7.

**Table 6:** Overall effect test for the acute effect of dynamic stretching on explosive power

	Studies	Heterogeneity test		Two-tailed test		MD(95%CI)
		I^2^	P	Z	P	
Random effects model	17	75.6	0.00	3.16	0.00	1.77(0.68, 2.87)

**Table 7:** Tests of the moderating effect of dynamic stretching on the acute effect of explosive power

Adjustment variables	Heterogeneity test		Category	Effect value&95%CI	Two-tailed test		Number of studies^**a**^
	I^2^	P			Z	P	
Stretching time	80.9	0.00	Less than 7min	0.44(-1.64, 2.52)	0.41	0.68	3
			7–10min	2.90(1.07, 4.73)	3.11	0.00	5
			More than 10min	1.40(-0.55, 3.35)	1.41	0.16	4
Average age	75.6	0.00	Less than 21years	1.95(0.20, 3.70)	2.18	0.03	8
			21–25years	1.39(-0.41, 3.20)	1.51	0.13	7
			More than 25years	2.52(-0.26, 5.30)	1.78	0.08	2
Subjects	77.2	0.00	athletes	1.67(0.31, 3.04)	2.40	0.02	10
			gymgoers	2.00(0.15, 3.85)	2.12	0.03	6
Sample size	75.6	0.00	Less than 20 cases	2.11(0.37, 3.84)	2.38	0.02	7
			21–30 cases	1.93(0.28, 3.59)	2.29	0.02	8
			More than 31 cases	0.07(-0.28, 2.23)	0.07	0.95	2
Years	75.6	0.00	2005–2010years	0.77(-0.73, 2.28)	1.01	0.31	7
			2011–2015years	2.53(0.89, 4.18)	3.01	0.003	7
			2016–2021years	1.55(-1.10, 4.21)	1.15	0.25	3
Quality of the literature	75.6	0.00	14–15 scores	0.28(-2.85, 3.40)	0.17	0.862	2
			16–17 scores	1.71(-0.31, 3.73)	1.66	0.10	7
			18–19 scores	2.02(0.47, 3.56)	2.56	0.009	8

^a^indicates that there are differences in the classification of some independent samples, resulting in a total number of studies with some moderating variables not equal to the total number of included studies

### Stretching time

A total of 12 studies were included in this conditioning group, and there was heterogeneity in the effect sizes of the three groups (I^2^ = 80.9%), indicating a moderating effect of stretching time on the acute effects of explosive force. The stretching time of 7–10 min had an effect size MD = 2.90 (95% CI (1.07, 4.73), *P* < 0.01), while the 95% CI for less than 7 min and more than 10 min passed the 0 point (*P* > 0.05), suggesting that the acute effect of stretching time of 7–10 min on explosive strength was the most significant.

### Mean age

A total of 17 studies were included in this conditioning group, and there was heterogeneity in effect size across the 3 groups (I^2^ = 75.6%), suggesting that there was some moderation of the effect size of age on the acute effect of explosive strength. The effect size MD = 1.95 (95% CI (0.20, 3.70), *P* < 0.05 for younger than 21 years of age, and 95% CI past 0 points, *P* > 0.05 for 21–25 years of age as well as older than 25 years of age), suggesting that the mean age of the study population was the most significant intervention effect for those younger than 21 years of age.

### Study population

A total of 16 studies were included in this conditioning group, and there was heterogeneity in effect size between the 2 groups (I^2^ = 77.2%), suggesting that there was some moderation of the acute effect of explosive strength with the effect size of the study subjects, with an effect size MD = 2.00 (95% CI (0.15, 3.85), *P* < 0.05 for bodybuilders and an effect size MD = 1.67, 95% CI (0.31, 3.04) for athletes, *P* < 0.05), which showing the most significant effect size for gym goers.

### Sample size

A total of 17 studies were included in this moderation group, and there was heterogeneity in the effect sizes of the 3 groups (I^2^ = 75.6%), suggesting that there was some moderation of the effect size of the sample size on the acute effect of explosive strength. The effect size MD = 2.11 (95% CI (0.37, 3.84), *P* < 0.05 for up to 20 cases, MD = 1.93, 95% CI (0.28, 3.59), *P* < 0.05 for 21–30 cases, and MD = 0.07, 95% CI past 0 points, *P* > 0.05). The results suggest that the sample size was most significant for sample sizes of less than 30 cases.

### Year of publication

The moderation group included a total of 17 studies in this moderation group, and there was heterogeneity in the effect sizes of the three groups (I^2^ = 75.6%), suggesting that there was some moderation of the effect size for year of publication on the acute effect of explosiveness (effect size MD = 2.53, 95% CI (0.89, 4.18), *P* < 0.05 for 2011–2015, and 95% CI for 2005–2010 and 2016–2021 were both past the 0 point, *P* > 0.05), showing that the effect size for 2011–2015 was the most significant.

### Quality of literature

The moderation group included a total of 17 studies in this moderation group, and there was heterogeneity in the effect sizes of the three groups (I^2^ = 75.6%), suggesting that there was some moderation of the effect size of literature quality on the acute effect of explosive force. (effect size MD = 2.02, 95% CI (0.47, 3.56), *P* < 0.01 for 18–19 scores, and *P* < 0.01 for 14–15 and 16–17 scores both had 95% CIs past the 0 point, *P* > 0.05), and the results suggest that the effect size for scores of 18–19 was the most significant.

## Discussion

This study explored the acute effects of different warm-up methods on lower limb explosive strength from an evidence-based medical perspective. The results of a net meta-analysis showed that SDS and DS were able to have a positive effect on explosive strength overall, while SS showed a negative effect, with a smaller but still statistically significant effect size and no results yet proving the effectiveness of other warm-up methods. This finding is largely in line with previous research findings [47, 48]. However, a recent meta-analysis was inconsistent with the results of this study [49], and the analysis suggests that this inconsistency might be related to the study methodology on the one hand. This study used a traditional meta-analysis, which was only able to analyse the relative validity of the effects of different warm-up methods on the acute effects of lower limb explosive strength, and it lacked a comprehensive comparative analysis between studies, whereas this study used a continuum analysis to test the stability and reliability of the relative evidence between different warm-up methods, and the indirect comparative modelling approach provided more accurate statistical analysis [50], which would allow relevant coaches and athletes alternatives to different warm-up methods, rather than in a traditional meta-analysis of individual warm-up method studies. Therefore, it is speculated that the study method might have influenced the results. Another aspect could be related to publication bias in the literature. Publication bias in meta-analyses can lead to lower actual effect sizes and can also increase the risk of no and negative effect sizes [51], so-called false 'positive' or false 'negative' results. The study did not test for the risk of publication bias, so there is a potential risk of publication bias. The study used a qualitative (funnel plot) test of bias to ensure the accuracy of the results to a certain extent.

### Analysis of the effect of different warm-up methods on the acute effect of lower limb explosive power

#### Dynamic stretching affects the effect

The results of the study showed that the DS group had a better effect size than the control group for both CMJ height(cm) [MD = 1.60, 95% CI: (0.67, 2.60)] and sprint time(s) [MD = -0.08, 95% CI: (-0.15, -0.008)], and the results of the SUCRA ranking chart indicated that, although slightly lower than the SDS group for CMJ height, its effect on explosive power was an acute effect that was significantly better than the other warm-up methods. Regarding the mechanism by which DS promotes explosive strength, some scholars believe that it might be related to DS increasing body and muscle temperature. During stretching, muscles actively contract and stretch, increasing temperature while decreasing viscosity; at the same time, increased muscle temperature can cause increased neuroreceptor sensitivity, and this increased sensitivity suggests that neuromuscles might show stronger motor unit activation through increased motor unit recruitment [52], thereby improving muscle contractile performance. For example, Fletcher et al. showed [53] that DS was able to significantly elevate muscle temperature and ultimately reverse longitudinal jump performance compared to SS. Furthermore, some scholars have found that the ability of DS to enhance subsequent explosive could be related to the preactivation of movement patterns [46, 54]. Studies have shown that it might stimulate the muscle shuttle to increase muscle reflex activity, thus allowing the muscle to better complete active contractions based on the characteristics of the subsequent movement and ultimately inducing a postactivation potentiation effect (PAP) [55].

DS has long been recommended as an essential component of warm-up activities, and its effects on improving joint mobility and preventing sports injuries are well established, but the effect on subsequent explosive power remains controversial, and the reasons for this controversy might be influenced by the duration of stretching, the study population and other factors. The results of the study showed significant heterogeneity (I^2^ = 75.6%) in the results of the comparison between the DS and control groups, suggesting that the variation caused by real differences in effect sizes accounted for 75.6% of the total variation, which can be considered a large degree of dispersion in the effect sizes of the individual studies; therefore, it is necessary to introduce moderating variables to investigate the heterogeneity in depth.

### In terms of stretch time

The test found that a stretch time of 7–10 min produced the largest effect size. This finding is largely in line with previous findings. A study by Mcmillian et al. [56] found that DS with a total time duration of 10 min was able to enhance subsequent jump performance compared to the SS and control groups. Similarly, Behm et al. [57] clearly suggested that a 7 min and 10 min DS could have a positive effect on subsequent explosive power. The reason for this finding might be that shorter periods of stretching do not make effective use of the compliance effects on tendon units, while longer periods can cause fatigue in the body, thus not maximising the benefits of dynamic stretching.

### Study population and mean age

In terms of the study population, fitness enthusiasts produced a larger effect size than athletes. The analysis suggests that athletes have already reached a higher level of physical performance as a result of undertaking long-term systematic training, whereas mass fitness participants are primarily interested in physical fitness, and therefore may have different needs and responses to dynamic stretching; in terms of average age, only the effect sizes under 21 years of age are significant. This age group is a sensitive period for neuromuscular development and is at the peak of natural growth in both speed and explosive ability [58], and therefore may produce a higher stimulatory response to dynamic stretching at this stage than in adulthood.

### In terms of sample size, years of publication and quality of literature

In terms of sample size, samples with fewer than 30 cases were the most significant, consistent with the ease of good results in trials with fewer subjects found in the meta-analysis by Kang Yujie et al. [59]. However, if the sample size is too small, potential errors due to random error factors cannot be excluded, regardless of whether the treatment effect survives as valid. Conversely, a sample size that is too large can result in a waste of resources. Therefore, the selection of the optimal sample size should consider both clinically significant and statistically significant differences in efficacy, as well as factors such as financial budget. In terms of the year of publication of the literature, the effect size of the studies between 2011 and 2015 was significant, consistent with the statement by Liu et al. [2] that "a large number of studies prior to 2016 demonstrated some facilitation of subsequent exercise performance by DS, after which many opposing views emerged that DS does not necessarily facilitate exercise performance". The reason for this outcome could be related to the relatively stable methodological quality of studies prior to 2016 [60]. In terms of the quality of the literature, only two of the 17 DS-related studies reported the use of blinding, which to some extent affects the quality of the literature, although a meta-analysis indicated that there was no significant correlation between the efficacy of physical methods and adequate blinding [61]. However, adherence to the 'blinding principle' can improve the internal consistency of trials and reduce bias due to the expectations of subjects, intervention implementers or outcome evaluation.

### Static stretching affects the effect

SS has become one of the most widely used warm-ups in sports due to its simplicity and controllability and low muscle damage [2]. Although SS can significantly improve joint mobility [62], its effect on subsequent explosive power must be further confirmed [63]. The results of this study showed that the effect size in terms of sprint time(s) was [MD = 0.07, 95% CI: (0.002, 0.13)], which was significantly different from the control group, and the effect size in terms of CMJ height(cm) was [MD = -0.75, 95% CI: (-1.70, 0.18)], which was not significantly different from the control group, but from the SUCRA ranking graph, it can be seen that the percentage of its area under the curve was 15.6%, which was much smaller than that of the control group, indicating that SS was able to negatively affect subsequent explosive. The reason for the lack of significant differences between SS and controls in terms of CMJ height could be that, compared to the complexity and coordination required for the short sprint, the measure of jumping ability is one dimensional; therefore, the effect of SS on relatively single-movement ability might be lower than that on relatively complex movement ability [64], and although the reverse vertical jump also requires a coordinated body effort, the time to complete the movement is relatively short, at least compared to the short sprint, and any change in the session might not have a significant effect on the outcome metrics.

Physiological and neurological studies have provided insight into the reasons for the negative impact of SS on explosive power. It has been found that prolonged static stretching of muscles affects the sensitivity of the muscle spindle (MS), which functions to encode information about the length of muscle extension as nerve impulses to the centre, reflexively generates and maintains muscle tension, and participates in the casual regulation of movement [65] through the coactivation of alpha-gamma motor neurons to ensure high sensitivity of the MS during muscle contraction [66]. However, prolonged SS can cause deactivation of the sensitivity of the γ system in MS, resulting in the inability of MS to transmit the actual length of the muscle to the superior centre, reducing the number of motor units excited out of neuroprotective inhibition and ultimately leading to a decrease in explosive power [67]. Another part of the study suggested that SS leads to a decrease in muscle–tendon unit (MTU) stiffness. The lower limb muscle–tendon union acts as a carrier of elastic energy storage and utilisation. In a state of constant muscle length, greater stiffness helps the muscle to generate more force during centripetal contraction [68]. Therefore, it has been suggested that SS might reduce muscle length and tone [69], thus preventing the muscle from being in an activated state, in turn leading to a decrease in stiffness [70] and ultimately having a negative impact on explosive power.

### Static combined with dynamic stretching to influence the effect

Since the strengths and weaknesses of SS are so obvious, it has been asked whether a combined approach of SS and DS could be used to take advantage of the improved joint mobility of SS while avoiding the detrimental effects of SS on explosive power through subsequent DS. The results of this study showed that, in terms of CMJ height(cm), the SDS effect size was [MD = 1.80, 95% CI: (0.43, 3.20)], which was significantly different from the control group; the effect size in terms of sprint time(s) was [MD = -0.06, 95% CI: (-0.16, 0.05)], suggesting that SDS can have some effect on subsequent CMJ height. No results were available to demonstrate the effect of SDS on sprint time. For the lack of significant differences between SDS and controls in terms of sprint time, the analysis suggests that this difference could be related to the number of included papers. Of the 35 included papers, only three [15, 29, 42] examined the effect of SDS on sprint time; in other words, the network comparison analysis was based on only these three papers, so the strength of the relevant evidence findings was significantly reduced. For example, only Chaouachi et al. [29] of the 3 papers showed no significant difference in SDS regarding subsequent sprint time because the study was conducted with physical education students, and the trained population was less susceptible to the acute effects of stretching than the untrained population, as confirmed by Egan et al. [71].

Regarding the mechanism by which SDS can enhance explosive, it has been suggested that it could be related to the reactivation of γ motor neurons in MS. Although the sensitivity of γ-motor neurons is inactivated after SS, the subsequent stimulation of the muscle shuttle by DS could "reawaken" the sensitivity of γ-motor neurons, and muscle contraction reverts to the coactivation pattern of α-γ-motor neurons [67], thus exploiting the advantages of SS and DS while avoiding their disadvantages. However, since few studies have been conducted, the results must be interpreted with caution, and further evaluation is needed.

### Other warm-up methods affect the results

In addition to the above warm-up methods, there are no results showing that foam axis rolling, PNF stretching and bouncy stretching can have effects on subsequent explosive power.

### Foam axis rolling

Also known as self-fascial relaxation, foam axis rolling is an emerging warm-up and relaxation technique in recent years. The practitioner uses self-weight to give the target muscle a certain amount of pressure to roll back and forth on the foam axis to improve the stretch and flexibility of the outer connective tissue of the muscle fibres [72], promote blood circulation, and increase myocyte oxygen and energy metabolism. The results of the present study remain largely consistent with previous studies and with a meta-analysis of FR by Wiewelhove et al. [49] showing that FR does not significantly affect subsequent explosive and that it is more suitable for postexercise relaxation than preexercise warm-up since it has been shown to be effective in relieving exercise-related muscle soreness. The few studies that have shown FR to significantly improve exercise performance suggested that FR could disrupt myofascial trigger-points (MTrP), which are nodules produced by transitional stress in skeletal muscle that can lead to muscle fatigue and stiffness [73], while Huang Haojie et al. [24] suggested that the stress produced by the foam axis on the muscle activated the Golgi Tdon organs (GTOs), which when active inhibit the muscle shuttle, causing a muscle relaxation response, a decrease in muscle tension, a decrease in muscle adhesion and an increase in muscle performance. Other studies have suggested that it could be related to the psychological factors of the participants since they believed that FR would improve their exercise performance [74].

### PNF stretching

The full name is proprioceptive neuromuscular facilitation (PNF), which first originated in the field of rehabilitation medicine for the treatment of diseases such as neuromuscular paralysis by activating the autonomic and cross-inhibitory effects of muscles to improve the function of specific muscles and later began to be widely used in the field of competitive sports, mostly used to prevent and treat sports injuries and improve joint mobility [75]. However, the stretching process requires the assistance of a professional to apply the force and is time-consuming and could be more suitable for professional athletes from a simple and economic point of view. The results of the MeSH meta-analysis showed that PNF stretching had a negative impact on explosive power, although the difference was not statistically significant, consistent with previous studies [76]. Therefore, Bradley et al. [77] suggested that PNF stretching should not be performed prior to explosive sports. The mechanism of the effect of PNF stretching on explosive strength has been considered by most scholars to be the same as that of static stretching and can be explained by affecting the sensitivity of sarcolemmal receptors.

### Stretching with elastic shock

BS has been gradually marginalised due to its special stretching mechanism – forcing the target muscle to elongate by means of rapid rebound, which most scholars believe causes a strong stretch reflex that in turn causes the muscle to contract to a shorter length than before the stretch and is therefore more likely to trigger muscle damage [78]. The results of the present study suggested that BS has a positive effect on subsequent explosive, although this effect was not statistically significant. Mariscal et al. [79] suggested that BS, by stimulating neuromuscular activity, could activate the stretch–shortening cycle (SSC), thereby improving subsequent sprint time. The outcome indicators included in this study, both CMJ in situ and short-distance sprinting, required centrifugal muscle elongation to store energy, followed by centripetal contraction to improve subsequent performance, which could, to some extent, explain why BS could have a positive effect on subsequent explosive.

### Limitations of the study

(1) The search of the literature for this study did not include unpublished literature, and some literature was not included due to the absence of a control group or incomplete data about outcome indicators, which might have affected the comprehensiveness of the information to some extent. (2) The small number of static combined with dynamic stretching and foam axis rolling related literature included might have weakened the argument to some extent. (3) Due to the warm-up method intervention short duration and other peculiarities, most studies did not use randomised, controlled studies but their own before-and-after controlled studies to avoid the influence of individual differences on the study results.

## Conclusion

(1) Static stretching reduces subsequent explosive, while dynamic stretching and static stretching combined with dynamic stretching are the two warm-up methods that significantly improve subsequent explosive, with dynamic stretching being the most stable and moderated by a variety of variables.

(2) The dynamic stretching time of 7–10min produced the best explosive, and the intervention effect was also influenced by modifying variables, such as study population and age.

(3) The quality and quantity of the included literature affects the overall effect of the intervention, and it is recommended that subsequent studies add high-quality studies based on strict adherence to the trial specifications.

## Data Availability

The datasets used and/or analysed during the current study is available from the corresponding author on reasonable request.
